# Colitis Promotes Adaptation of an Intestinal Nematode: A *Heligmosomoides Polygyrus* Mouse Model System

**DOI:** 10.1371/journal.pone.0078034

**Published:** 2013-10-22

**Authors:** Katarzyna Donskow-Łysoniewska, Justyna Bien, Klaudia Brodaczewska, Katarzyna Krawczak, Maria Doligalska

**Affiliations:** 1 Department of Parasitology, University of Warsaw, Warsaw, Poland; 2 Witold Stefanski Institute of Parasitology, Polish Academy of Sciences, Warsaw, Poland; National Institutes of Health, United States of America

## Abstract

The precise mechanism of the very effective therapeutic effect of gastrointestinal nematodes on some autoimmune diseases is not clearly understood and is currently being intensively investigated. Treatment with living helminths has been initiated to reverse intestinal immune-mediated diseases in humans. However, little attention has been paid to the phenotype of nematodes in the IBD-affected gut and the consequences of nematode adaptation. In the present study, exposure of *Heligmosomoides polygyrus* larvae to the changed cytokine *milieu* of the intestine during colitis reduced inflammation in an experimental model of dextran sulphate sodium (DSS)- induced colitis, but increased nematode establishment in the moderate-responder BALB/c mouse strain. We used mass spectrometry in combination with two-dimensional Western blotting to determine changes in protein expression and changes in nematode antigens recognized by IgG1 in mice with colitis. We show that nematode larvae immunogenicity is changed by colitis as soon as 6 days post-infection; IgG1 did not recognize highly conserved proteins Lev-11 (isoform 1 of tropomyosin α1 chain), actin-4 isoform or FTT-2 isoform a (14-3-3 family) protein. These results indicate that changes in the small intestine provoked by colitis directly influence the nematode proteome. The unrecognized proteins seem to be key antigenic epitopes able to induce protective immune responses. The proteome changes were associated with weak immune recognition and increased larval adaptation and worm growth, altered localization in the intestine and increased survival of males but reduced worm fecundity. In this report, the mechanisms influencing nematode survival and the consequences of changed immunogenicity that reflect the immune response at the site colonized by the parasite in mice with colitis are described. The results are relevant to the use of live parasites to ameliorate IBD.

## Introduction

Nematodes suppress the immunity generated by infection and also affect responses to other non-nematode antigens [1]. Some studies have shown that autoimmune diseases are increasing in prevalence in areas where exposure to helminths is rare. These observations suggest that the loss of pathogens and parasites removes a natural governor that helps to prevent disease due to immune regulation [2]. Epidemiological and laboratory studies confirm that nematodes prevent immune-mediated diseases. The immunological mechanism underlying the local therapeutic effect of gastrointestinal nematodes on inflammatory bowel diseases and on different inflammatory tissue is not clearly understood and is currently being intensively investigated. It was previously suggested that proteins released from nematodes suppress activation of the Th1 inflammatory response in the inflammatory tissue not simply through modulation of the Th2 response but also by mechanisms dependent on macrophages [3,4].

Therapy with living nematodes seems to be the most effective therapy. It has been argued that treatment of patients with living nematodes has disadvantages and in order to survive in an adverse and aggressive environment, the nematodes secrete several soluble factors that interact with host cells and may modify host-cell homeostasis [5,6]. However, little attention has been paid to the basic physiological mechanisms for protecting the parasite against an excessive inflammatory response and the consequences for nematode survival during therapy.

The development of immunologically well-defined laboratory models of intestinal nematode infection has allowed substantial advances to be made in understanding the immunological basis of the effector mechanisms operating during infection under controlled laboratory conditions. The *H. polygyrus*-mouse system is widely used for studies of parasite immunomodulation in inflammatory diseases for several reasons. Nematodes cause a chronic, asymptomatic gastrointestinal infection, which is very similar to those observed in hookworm *Necator americanus* infection in man [7]. Primary exposure of mice to the L4 stage reduces inflammation in an experimental model of dextran sulphate sodium (DSS)-induced colitis by leukocytes, especially macrophage infiltration into the small intestine and inhibition of those in the colon [4]. A possible mechanism of inhibited recruitment of monocytes into the inflamed colon mucosa in the presence of nematodes has been described [4]. Interestingly, in this study we detected that the changes in the small intestinal cytokine *milieu* induced by *H. polygyrus* larvae enhanced nematode survival and increased L4 establishment in BALB/c mice with colitis.

During *H. polygyrus* infection, L3 larvae move to the small intestine and localise in the small intestinal walls by day 3 [8]. L4 larvae reside between the two muscle layers in the *muscularis externa* and are able to sit unharmed in the gut walls in this location in immune-competent mice despite the intense granuloma developed around them in a state resembling arrested development [9,10]. Developmental pathways are initiated by host-specific signals and lead to the maturation of larvae into adult parasites. The molecular details of this process are still unknown. The recognition of L4 antigens is strictly associated with high production of specific IgG1 and IL-4 [11]. Depending on the intensity observed in different strains of mice, the immune system can control the initial establishment of infective larvae, regulate their development and influence the survival, fecundity and clearance of the mature stages, but still little is known about the specific antibody response during infection and how the host immune response influences worm fitness.

In this study, we analysed the consequences of colitis on L4 and adult nematodes. We show that the colitis-affected gut changed the *H. polygyrus* proteome as soon as 6 days post-infection. We describe changes in the small intestine provoked by nematode therapy and the fitness of L4, adult worms and 2^nd^ generation larvae. We used mass spectrometry in combination with two-dimensional Western blotting to determine changes in the immunogenic antigens recognized by specific IgG1 antibody. The results indicate that the colitis-affected gut may support parasite survival and treatment with live nematodes might have unintended and adverse effects on the host. 

## Materials and Methods

### Ethics statement

All experimental procedures were performed according to the Polish Law on Animal Experimentation and Directive 2010/63/UE and approved by the First Warsaw Local Ethics Committee for Animal Experimentation with the approval ID 151/2011. 

### Animals

The experiments were conducted in the BALB/c strain of mice, which is an intermediate responder to *H. polygyrus* infection [11]. Pathogen-free males were 8 weeks old and weighed 22-27g at the start of the study. Mice were allowed to adjust to the laboratory conditions for 7 days before experimental manipulation at the animal-house facilities at the Faculty of Biology and placed in groups of five in cages in a controlled room with temperature 24-25°C, humidity 50% and lighting regime of 12 h/12 h cycle, and were allowed *ad libitum* access to drink and commercial pellet food. All experiments and tests were performed at least in triplicate to ensure accurate results and the results of one representative experiment are shown. 

### Induction of DSS-induced colitis and infection with H. polygyrus

For the induction of acute colitis, mice received 5% dextran sulphate sodium (DSS) a sulphated polymer, 35–50 kDa (ICN Biomedicals Inc., OH, USA), in drinking water for 3 days before oral infection with 300 L3 *H. polygyrus* until the end of the experiment. Fresh DSS solution was prepared every second day. L3 for the infection were collected from the same faecal culture at the same time.

Induction of colitis was determined by the clinical symptoms: body weight, stool consistency, faecal bleeding and diarrhoea [4]. Daily clinical assessment of animals included measurement of body weight, the presence of blood in the stools by a paper test (Beckman Coulter Inc., Fullerton, CA) and evaluation of stool consistency by the same researcher. Body weight changes were calculated by subtracting the starting weight (at day 1) from the actual weight on a specified day and expressed as a change in grams from day 1. Stool consistency (diarrhea score) and fecal blood was scored separately on a scale 0-2. Loose stool was defined as the formation of a stool that readily became paste upon handing (1). Diarrhea was defined as no stool formation (2). Fecal blood was defined as slightly bloody (1) and bloody (2). Significant differences were not detected in the daily consumption of water and water with DSS between groups of mice. 

Five mice per group were sacrificed at 6 and 15 days post-infection (DPI). These mice were killed by increasing CO_2_ concentration. 

### Preparation of small intestine

The small intestines were removed, opened longitudinally and washed in ice-cold physiological phosphate-buffered saline PBS pH 7.4 without calcium and magnesium. The mucosal layer was separated by careful scraping with a glass slide. The homogenate with a cocktail of protease inhibitors (Roche Diagnostics Ltd, Mannheim, Germany) was centrifuged at 4.000g at 4°C for 45 min. The supernatant was stored at -80°C prior to cytokine and antibody analysis.

For the immunohistological analysis at 6 DPI, 1-cm sections of the small intestine were taken 5 cm proximal to the pylorus, frozen in liquid nitrogen and stored at -80°C. Eight-μm-thick consecutive frozen sections were prepared. Intestine **s**ections were stained with haematoxylin and eosin (H&E) according to standard procedures for light microscopic examination (OLYMPUS BX50, Tokyo, Japan). To quantify the numerical densities of leukocytes in the small intestine, images of each section were analysed using a computer. In each case, H&E sections from three intestine tissues of five mice per group were counted. The results are expressed as the number of cells per field of view.

### Cytokine and antibody detection

Cytokine levels were titrated using the ELISA method. IL-2, IL-12 and MCP-1 concentrations were measured by ELISA using monoclonal antibodies according to the manufacturer’s guidelines (BD Biosciences, Pharmingen, San Diego, CA, USA). IL-22, IL-17A, IL-10, IL-6 and TGF-β were measured using monoclonal antibodies according to the manufacturer's guidelines (e-Bioscences, San Diego, USA). For the TGF-β measurement, the samples were acidified. Latent and active cytokine excreted into the culture medium was measured in each sample. The plates were read at 450 nm using u-Quant (BD, Costar, Acton, MA, USA). The mean optical densities (OD) of triplicate cultures were compared with the standard curves prepared using recombinant cytokines. The detection limit of the assays was 2pg/mL for IL-6, 8pg ⁄mL for IL-22, 4pg ⁄mL for IL-17A, 2pg/mL for IL-2, 30pg/mL for IL-10 and 8pg/mL for TGF-β, 2pg/mL for IL-12 and 4ng/mL for MCP-1.

Mucus IgG1, IgA and IgE responses to L4 and adult antigen were measured in individual mice. Maxisorb microtitre plate wells (Costar, Acton, MA, USA) were coated overnight at 4°C with 100 μL L4 somatic antigen in 50mM carbonate buffer, pH 9.6. The plates were washed and blocked with 5% non-fat milk powder in PBS pH 7.4 for 1h at room temperature (RT). After washing, 50μl of abomasal mucus sample, diluted 1:5, was added and incubated for 2h at RT. Wells were re-washed and 50μL of goat anti-mouse IgG-horseradish peroxidase (HRP) (Santa Cruz Biotechnology, 1:20000)/Anti-Mouse IgA (α-chain-specific)-HRP (Sigma, 1:200)/rat anti-mouse IgE (Serotec, Oxford, UK; 1:2000) and HRP-conjugated polyclonal rabbit were added for 1h at RT. After the final wash, TMB substrate was added. Reactions were stopped by 2M sulphuric acid and the OD values were read at 490 nm.

### Parasite and burden

Six DPI, tissue dwelling *H. polygyrus* larvae were counted *in situ* in 2-cm intervals along the small intestine. The mean larval position was calculated as (number of larvae per segment x distance of segment from stomach) divided by (total larvae x intestine length). Fourth-stage larvae were counted [12]. The small intestine of each infected mouse was removed, ligated at both ends with cotton twine to prevent contamination of the medium with digested matter and incubated for 2h at 37°C in Petri dishes containing 100μL RPMI 1640 Medium (Gibco, Paisley, UK) with 10% Glutamax (Gibco, Paisley, UK). The larvae were harvested and counted from each individual mouse. 

For samples taken 15 DPI, adult worm numbers were estimated using the Baermann technique [13]. 

Faecal samples were collected separately from five mice in each group, faecal egg counts were measured and the number of eggs per gram (EPG) of faeces was calculated.

Total body length of 20 male and 20 female worms per mouse for L4 and adults were measured to the nearest 1μm using a dissecting light microscope at x40 magnification fitted with an ocular micrometer. Each worm was straightened in a drop of RPMI 1640 medium and was assessed morphologically. Sex of L4 larvae was determined by the presence of bursa at the caudal end of male larvae. For all stages, sex ratios were calculated by dividing the number of male by the number of female parasites.

### Adult female reproduction *in vitro*

Five females from each mouse were placed individually into wells of a 24-well plate (Costar, Acton, MA, USA) containing 500µL RPMI 1640 supplemented with 100U of penicillin/streptomycin per mL (Gibco, Paisley, UK) and incubated at 37°C and 5% CO_2_. After 24 hours, each worm was removed to the fresh medium. The number of eggs per female from the first 24h (0-24h) and the next 24h (24-48h) were counted.

### *H. polygyrus* larvae culture *in vitro*

Eggs from the 24–48h *in vitro* culture were washed five times in PBS (pH 7.2), counted and 500 eggs were placed in the wells of a plastic culture containing 5mL of Nematode Growth Medium (NGM) agar [14] with *Escherichia coli* strain OP50. The viability of eggs was estimated by trypan blue staining and was found to be at least 92%. Eggs were left in the dark at 21°C. After 24h, unhatched eggs or free first-stage larvae (L1) were observed. Second-stage larvae (L2) were observed after 72h and third-stage larvae (L3) after 4 days. After 2 days and 10 days, L1 and L3 stage respectively were harvested, assessed morphologically and the number of the larvae was evaluated microscopically. 

### Direct effects of DSS on worms

To exclude the direct influence of DSS on worms, L4 and adults of *H. polygyrus* from both groups were cultured *in vitro*. Hundred early L4 larvae or five females were incubated in a 24-well plate containing 500µL RPMI 1640 supplemented with 100U of penicillin/streptomycin per mL alone, or in medium containing 0.5%, 2%, 5% and 10% DSS for 72h. The impact of *in vitro* exposure to graded doses of DSS on L4 and adult worm survival, egg production by adults and egg hatching was studied as described above.

### Larvae somatic extract preparation

Five hundred L4 stage from control mice, DSS-treated mice and from *in vitro* culture with DSS were sonicated in 0.5mL PBS (7.2) and centrifuged 15 min at 10.000g. The solution was sterilized using a 0.22-μm filter (Millipore, Carrigtwohill, Ireland). The final protein concentration of L4 homogenate was measured by the Bradford technique. Antigen containing <20 endotoxin units/mg protein was collected and stored at -80°C until use. 

### Gel electrophoresis

For 1D electrophoresis, protein samples of L4 somatic extracts were boiled for 10 min in 2% sodium dodecyl sulphate (SDS, Sigma) with 5% β-mercaptoethanol (Sigma) and centrifuged for 10 minutes at 15.000g. 10μg of each sample were separated on on 12% SDS polyacrylamide gels for 40 min at a constant 200 V using a Bio-Rad Minigel System (Bio-Rad Laboratories, Richmond). Gels were silver stained using PlusOne™ Silver Staining kit (Amersham Pharmacia, Uppsala, Sweden) or proteins were transferred onto nitrocellulose membrane. 

For 2D electrophoresis, the soluble protein extracts of L4 were homogenized in a ground-glass hand-held homogeniser in lysis buffer [8M urea, 40mM Tris base, 4% CHAPS] supplemented with a cocktail of protease inhibitors (Roche), followed by centrifugation at 13.000g for 5 min. The supernatant was collected and purified using a 2D Clean-Up Kit (GE Healthcare). The protein concentration was determined using a NanoDrop ND1000. 

Isoelectric focusing was performed using IPG strips and a Protean IEF Cell. 30μg of L4 protein in rehydration buffer was actively loaded onto 7cm pH 3–10 immobilized pH gradient (IPG) strips at 250V for 15 min, followed by 4.000V at 20°C and a maximum current setting of 50μA per strip. Focused strips were reduced and alkylated by 25 min incubation in equilibration buffer (50mM Tris-HCl, 6M urea, 2% SDS, 30% glycerol, 5mM tributylphosphine and bromophenol blue). Equilibrated proteins were then separated in the second dimension on SDS-PAGE in a Dodeca Cell (Bio-Rad) at 200V for 55 min. Gels were visualized using silver stain or used for Western blotting. Images were analysed by ImageMaster^TM^ 2D Platinum v6.0 (GE Healthcare, Uppsala, Sweden).

### Immune detection

Immune serum was obtained from six mice infected with 300 L3 of *H. polygyrus*; inoculation was performed three times during two months. After 2 weeks of each inoculation, mice were treated with anthelmintic (Pyrantelum, Cobantril; Pfize) and after 1 week the procedure was repeated. Serum was prepared from blood samples taken after cardiac puncture.

Proteins from 1D and 2D gels were transferred onto nitrocellulose membranes (Bio-Rad Laboratories) in cold transfer buffer (25mM Tris, 192mM glycine, 20% (v/v) methanol pH 8.3) at 100V for 30 min using a semi-dry blotting apparatus (Bio-Rad Laboratories). The membranes were blocked overnight in 5% skimmed milk in Tris-buffered saline/0.1% Tween 20 (TBS-T) at 4°C then exposed to sera from experimentally *H. polygyrus*-infected mouse (1:100) followed by mouse IgG conjugated to HRP (Santa Cruz Biotechnology, 1:20000). Samples without primary antibody were used as negative controls. The 1D immunoblot was developed with 3,3’-diaminobenzine (DAB, Sigma-Aldrich, Steinheim, Germany) and developed until the optimum colour was obtained. The 2-DE blots were visualized by enhanced chemiluminescence (SuperSignal West Pico Chemiluminescent Substrate, Pierce) by exposing the filters to X-ray film. The enhanced chemiluminescent reaction was developed according to the manufacturer’s instructions with X-ray films exposed to the blots. The immunoreactive spots on 2-DE Western blot were matched to their homologues in 2-DE silver-stained gels. The spot volume was used as the analysis parameter for quantifying protein expression with Bio-Rad Quantity One software (Hercules, CA, USA). 

### Mass spectrometry and bioinformatics

Tandem mass spectrometry was carried out. Briefly, spots of interest that were recognized by IgG1 were excised from the 2D gels using sterile disposable scalpel blades then subjected to trypsin digestion. Gel pieces were washed three times in 100ml of 50mM ammonium bicarbonate, 50% (v/v) methanol and then twice in 100ml of 75% (v/v) acetonitrile, before drying. Gel pieces were rehydrated with trypsin solution (20mg trypsin/ml 20mM ammonium bicarbonate), and incubated for 4h at 37°C. Peptides were extracted from the gel pieces by washing twice in 100μL of 50% (v/v) acetonitrile/0.1% (v/v) trifluoroacetic acid, before being transferred in solution to a fresh 96-well plate and dried before mass spectrometry analysis. All peptide samples were separated on an LC system (Famos/Switchos/Ultimate, LC Packings) using water that contained 0.1% TFA as the mobile phase and then transferred to a nano-HPLC RP-18 column (nanoACQUITY UPLC BEHC18; Waters Associates, Milford, MA, USA) using an acetonitrile gradient (0–60% ACN) in the presence of 0.05% formic acid with a flow rate of 150μL/min and analysed by electrospray ionization (ESI) Orbitrap mass spectrometry. A blank run preceded each analysis.

Tandem mass spectral data was carried out using the MASCOT program (Matrix Science Ltd, v2.1.1, London, UK) against the NCBI and wormBase databases. For gel spot identifications, a peptide mass tolerance of 0.1Da was used. 

### HPLC analysis of L4 antigen

High-pressure liquid chromatography was performed on a ProteinPak column (7.5mm X 300mm; Waters Associates) using the HPLC Alliance 2695 coupled to a photodiode array detector (Waters Associates). A total of 100µL of antigen solution was loaded onto the column and eluted isocratically PBS (pH 7.4) with a flow rate of 400μL/min for 45 min. Spectra were collected in the range 190–650nm. HPLC fractioning experiments were calibrated with synthetic peptides to allow comparisons between experiments. Data was analysed with the Empower program (Waters Associates). Representative chromatograms of analysis at 254nm spectra at chosen time points are shown.

### Statistical analyses

The data were collected from three independent experiments. The results and statistical evaluation of a representative experiment are presented. The significance of differences between groups was determined by analysis of variance (ANOVA) using MINITAB Software (Minitab Inc., PA, USA). Wherever appropriate, the Chi-square test (http://www.graphpad.com/quickcalcs/index.cfm) was used to test deviation from ratios predicted by random occurrence. All values are expressed as mean ± SE. A P-value <0.05 was considered to be statistically significant.

## Results

### Clinical symptoms and small intestine changes

**Figure 1:**
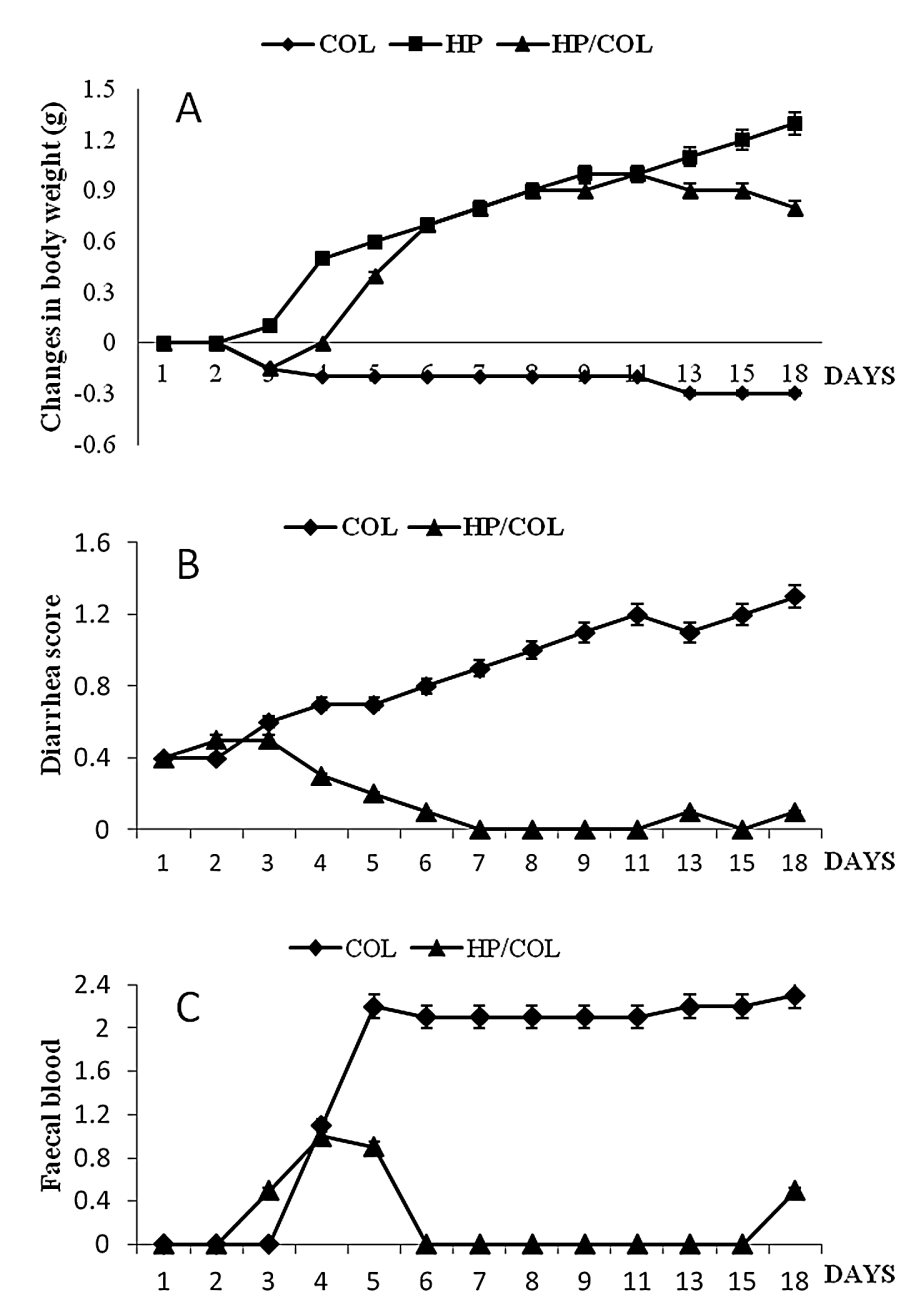
Effect of *H. polygyrus* infection on colitis symptoms; weight change expressed as a change in grams from day 1 (A), diarrhea score as an indicator of stool consistency (B), presence of blood in the feces (C). BALB/c mice were treated with 5% dextran sulfate sodium (DSS) in drinking water for 3 days before oral infection with 300 of infective L3 larvae *H. polygyrus* until the end of the experiment. Data were analyzed by one-way ANOVA using MINITAB Software. Data are presented as the mean values ± SE.

*H. polygyrus* infection reversed clinical symptoms in mice treated with DSS. Mice infected with worms and treated with DSS did not develop clinical symptoms during the 5 days of the experiments and 2 days after infection, as previously reported (Figure 1). 

**Figure 2:**
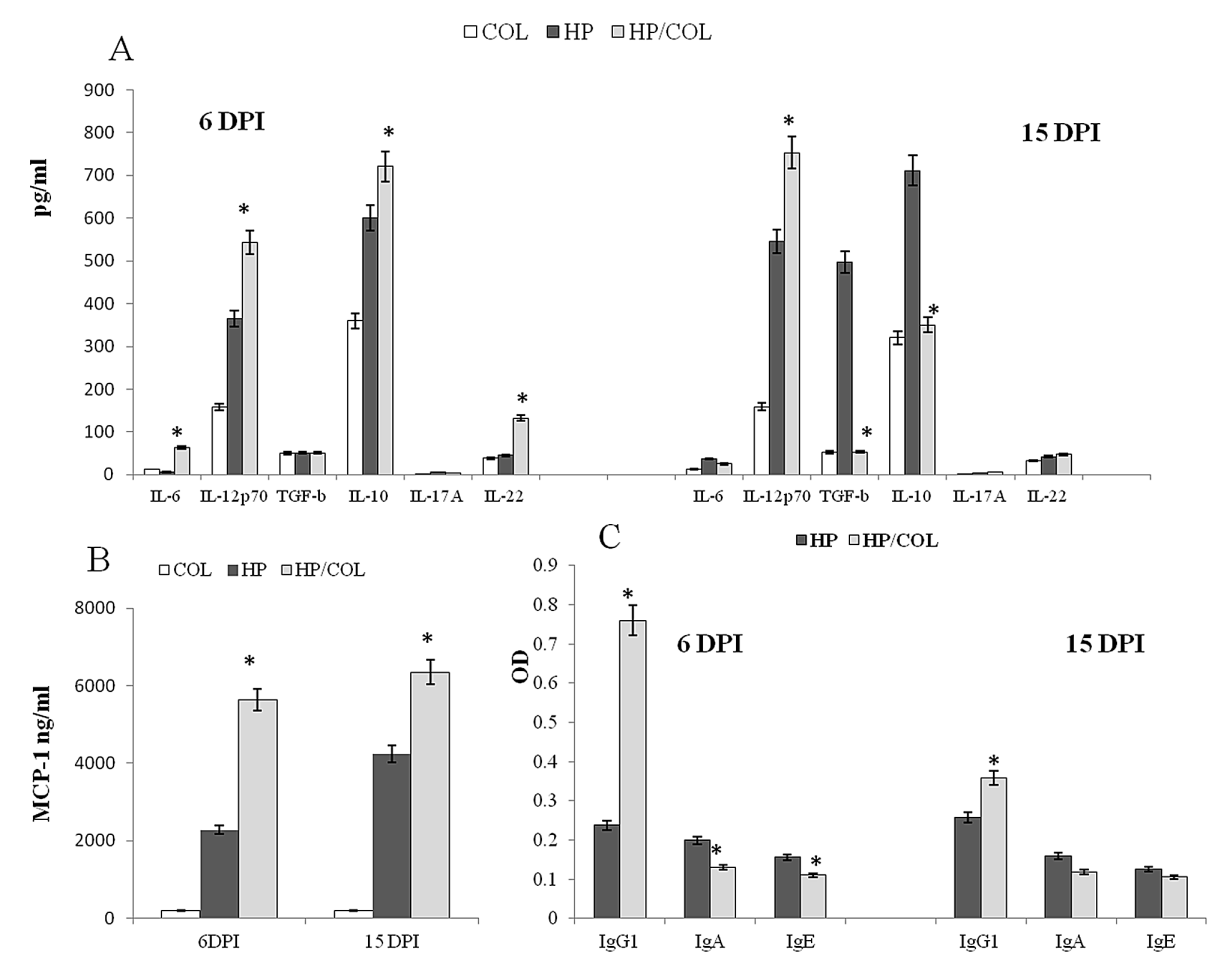
Effect of DSS and/or *H. polygyrus* infection on IL-12p70, IL-6, IL-22, IL-17A, IL-10, TGF-β (pg/mL, A) and MCP-1 concentration (ng/ml, B) in the small intestine at 6 and 15 days post infection with *H. polygyrus* and mean absorbance (OD) of intestinal mucus IgG1, IgA and IgE against somatic *H. polygyrus* L4 and adults (C). The concentration of cytokines in the small intestine of mice treated for colitis with dextran sulphate sodium (COL); infected with *H. polygyrus* (HP) or treated for colitis and infected with *H. polygyrus* (HP/COL) and the specific antibodies levels were measured by ELISA. The results are expressed as the means ± SE of five mice. Statistical significance between groups was assessed by ANOVA; *P < 0.05 compared to values obtained in the small intestine of control untreated mice infected with *H. polygyrus* (HP).

Concentration of cytokines was measured *ex vivo*, in the scraped mucosa at 6 and 15 DPI (Figure 2A, B). Mice with colitis infected with *H. polygyrus* had higher concentrations of IL-6, IL-12p70, IL-10, IL-22 and MCP-1 but lower amounts of IL-17A (from 5.4 pg/mL to 3.2 pg/mL) at 6 DPI. At 15 DPI, in mice treated with DSS and infected with *H. polygyrus*, production of IL-12p70 and MCP-1 was higher while concentration of IL-6, TGF-β and IL-10 was significantly lower. 

The concentration of specific IgG1 in the small intestine to L4 and adult worms was higher in mice with colitis than untreated mice (Figure 2B). The level of IgG1 specific to L4 at 6 DPI increased threefold. The concentration of IgA and IgE to L4 at 6 DPI and to adults at 15 DPI was partly reduced and there were no significant differences in the concentration of antibodies in the serum at 6 and 15 DPI between these two groups of mice. IgG1 specific to L4 was not detected in the small intestine mucosa of naïve mice or mice with colitis without nematode infection (negative controls; data not shown).

**Figure 3:**
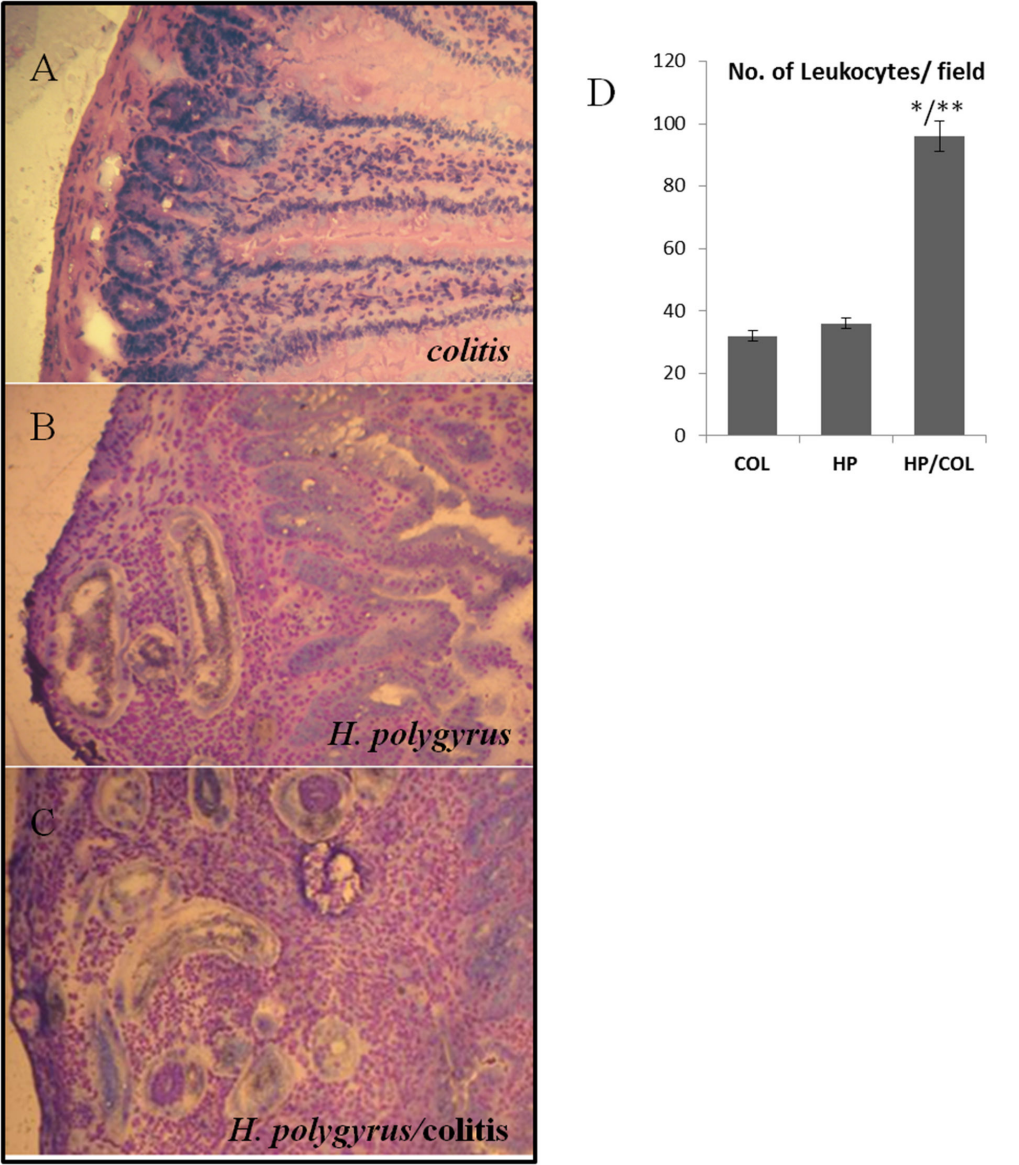
Light micrograph of haematoxylin and eosin (H&E, original magnification x 40) staining of mouse small intestine of BALB/c mice with colitis (A) or/and infected with *H. polygyrus* (B, C) on day 6 post-infection. Quantification of the number of leukocytes per field of the small intestine (D). Eight-micrometer sections of frozen intestinal tissue were cut, fixed and stained with H&E. Results are representative of three experiments each with five mice per group. Data are shown with the standard deviation. Statistical significance between groups was assessed by ANOVA; *P < 0.05 compared to values obtained in the small intestine of untreated mice infected with *H*. *polygyrus* (HP); **P < 0.05 compared to values obtained in the small intestine of mice with colitis (COL).

H&E staining of frozen sections confirmed the changes in the small intestine at 6 DPI. *H. polygyrus* L4 caused increased cellular infiltration into the mucosa and submucosa of the small intestine of mice treated with DSS (Figure 3). 

Quantification of the number of leukocytes per section in the small intestine confirmed an inflammation in the small intestine (Figure 3B). There were significantly more cells infiltrating the small intestine of mice with colitis infected with *H. polygyrus* L4 than cells infiltrating the small intestine of mice with DSS treatment or *H. polygyrus* infection. 

### Worm establishment

**Figure 4:**
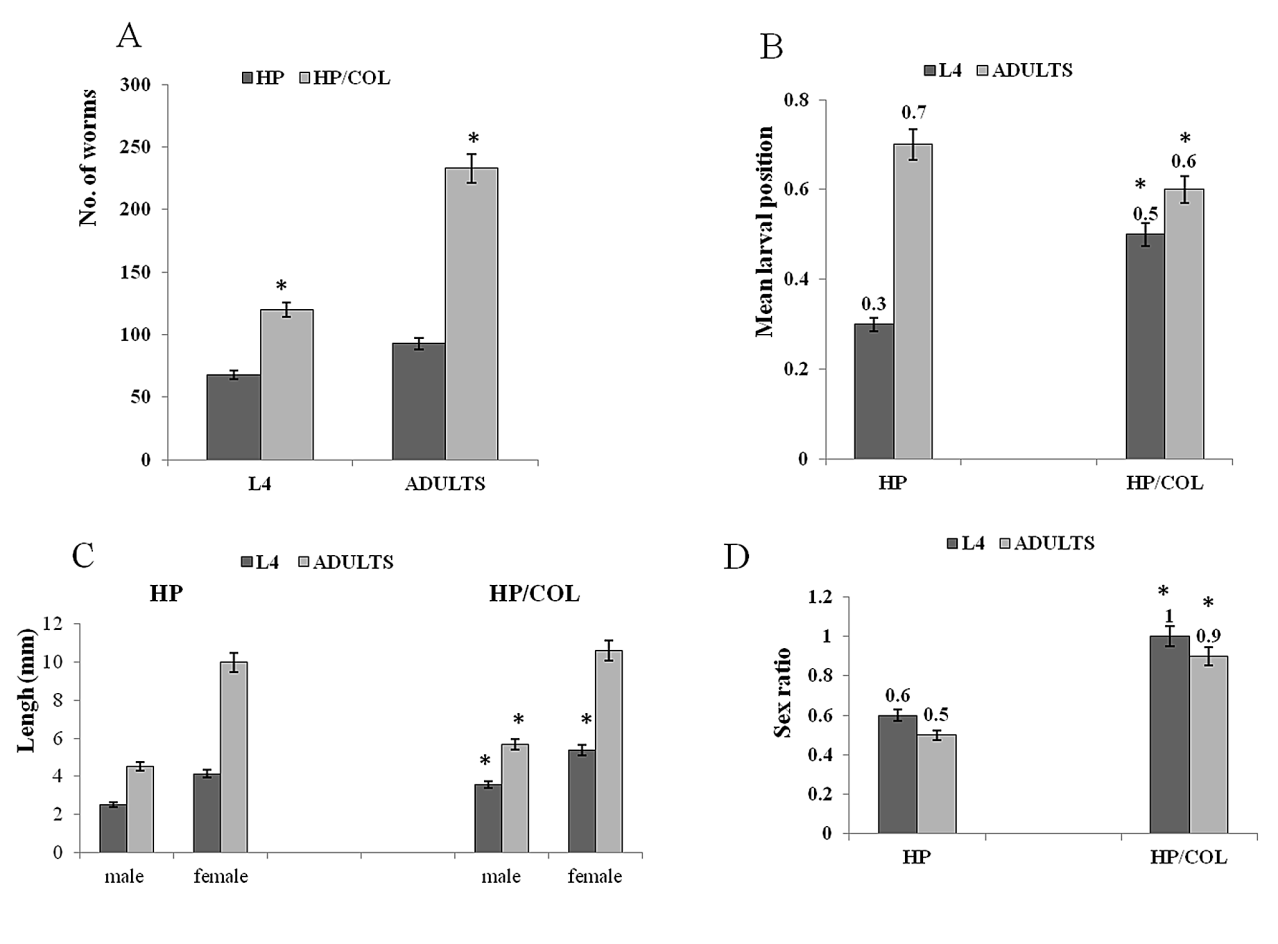
The consequences of colitis in *H. polygyrus* nematodes. A. The mean number of L4 and adult worms isolated from mice with colitis infected with *H. polygyrus* (HP/COL) and from control infection (HP). B. The mean worm position (fraction of intestine length). C. Mean worm length of male and female L4, and adult *H. polygyrus* (mm). D. Sex ratio (male: female; P<0.001 Chi-square test); BALB/c mice were orally infected with 300 *H. polygyrus* L3, 3 days after DSS treatment. The mice were sacrificed at day 6 and 15 post infection. Tissue –dwelling larvae and adults were counted *in* *situ* to determine the total number and distribution along small intestine. Individuals were removed, sexed and measured. The mean larval position was calculated as (number of larvae per segment x distance of segment from stomach) divided by (total larvae x intestine length). Each data point represents the means ± SE of ﬁve mice. *P < 0.05 comparing to the level of control mice infected with *H. polygyrus*.

BALB/c mice were infected with 300 *H. polygyrus* L3 stage and sacrificed 6 and 15 days later at a time when the L4 larvae occupied the submucosal tissue near the muscularis or the small intestine mucous surface respectively. Larvae were counted *in situ* and their distribution across the length of the small intestine was determined as the mean larval position (Figure 4B). Individual larvae and adults were extracted and their length as an indicator of development was measured. Lengths are presented separately for each sex (Figure 4C). 

The number of L4 and adult stages was significantly enhanced in mice with colitis compared with untreated mice (Figure 4A). There was no change in the morphology of worms. Freshly collected worms of both groups were bright red in colour due to the haemoglobin in the cuticle body wall, and pseudoceolomic fluid of the parasite. Adult worms had a typical coiled and corkscrew appearance.

Larvae in control mice clustered in the duodenum whereas larvae in mice with colitis invaded more distal regions of the small intestine. The distribution of adults in the small intestine was not significantly inﬂuenced by colitis (Figure 4B). 

Colitis affected worm length (Figure 4C). Adult males and larvae of each sex were significantly longer in mice with colitis than control mice. Colitis had a significant effect on the sex ratio of L4 and adult *H. polygyrus*. The sex ratio from colitis mice of 1.0 and 0.9 for L4 and adults, respectively, was 40% more than the sex ratios of 0.6 for L4 and 0.5 for adult *H. polygyrus* worms from control mice. The sex ratio of worms from mice with colitis with a value 0.9–1 reflected equal survival of males and females.

### Effect of colitis on the next generation of nematodes

**Figure 5:**
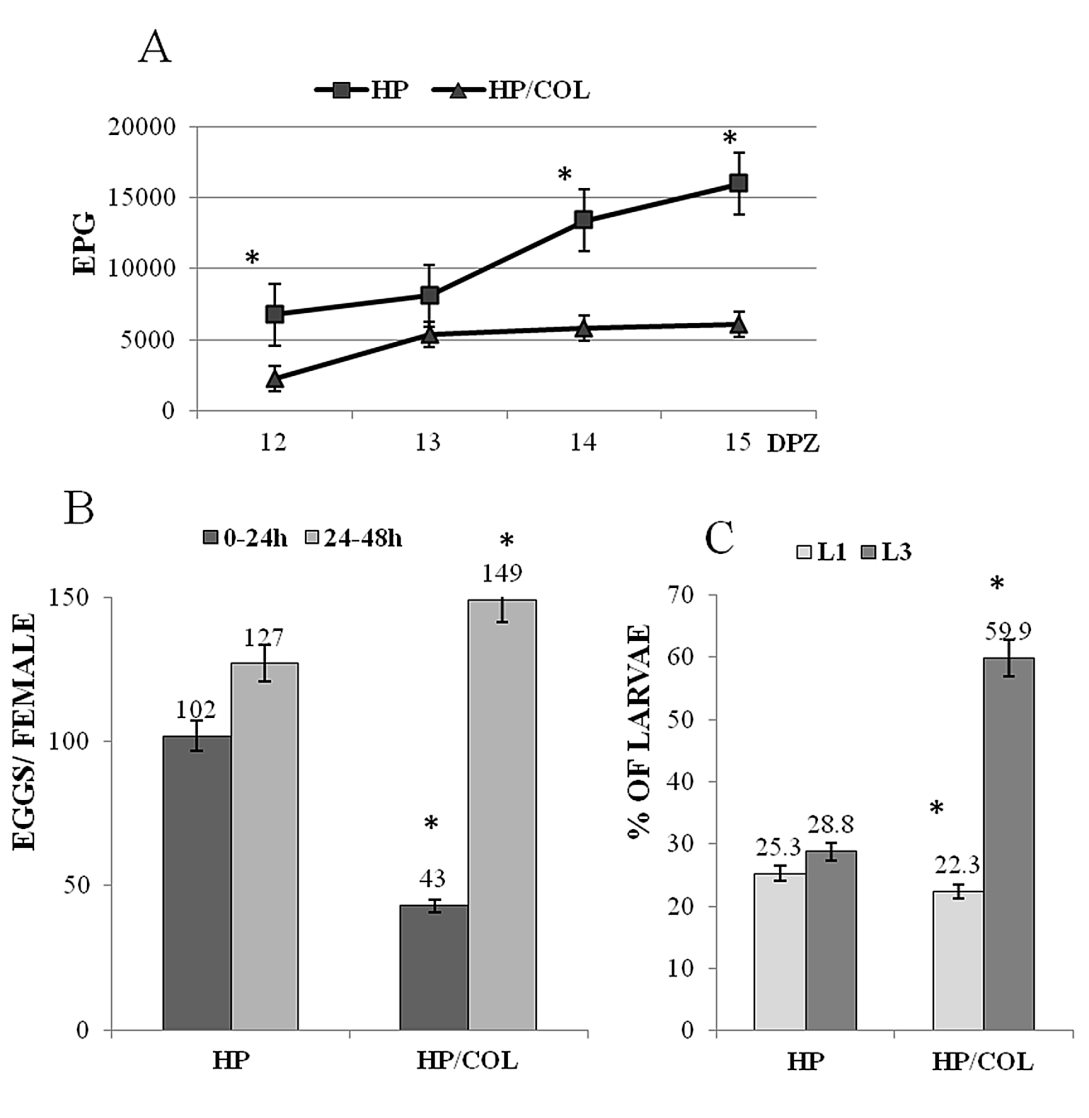
The consequences of colitis on female *H. polygyrus* egg production and the next generation of larvae. A. *H. polygyrus* egg production per gram of faecal (EPG). B. Mean *in* *vitro* worm egg production for 1 female during first 24 hours (0-24) and next 24 hours (24-48). C. The percentage of next generation L1 and L3 stage in *in* *vitro* agar culture. Eggs were counted in the feacal samples and in the *in* *vitro* culture. The eggs from the *in* *vitro* culture were cultured on the Nematode Growth Medium agar with *E. coli* strain OP50. After 3 days L1 and after 10 days L3 stage were harvested and counted. HP- control infection, HP/COL-infection of mice with colitis. Each data point represents the means ± SE of ﬁve mice. *P < 0.05 comparing to the results derived from nematodes isolated from mice with colitis.

Nematodes in mice with colitis had a significantly lower egg output per gram of faeces than the nematodes from the control infection on days 12, 13, 14 and 15 (Figure 5A). The number of eggs produced *in vitro* by female worms harvested from mice at 15 DPI during the first 24 hours (0–24h) confirmed the results obtained *in vivo*. However, during the next 24 hours (24–48h) the same females isolated from mice with colitis produced significantly more eggs than nematodes harvested from control mice (Figure 5B). 

The treatment of mice with DSS slightly delayed egg hatching measured as a L1 number but there twice as many L3 larvae was harvested from mice with colitis compared to control mice (Figure 5C). The morphology of larvae in these two groups of mice was not affected.

### Direct effects of DSS on worms

**Figure 6:**
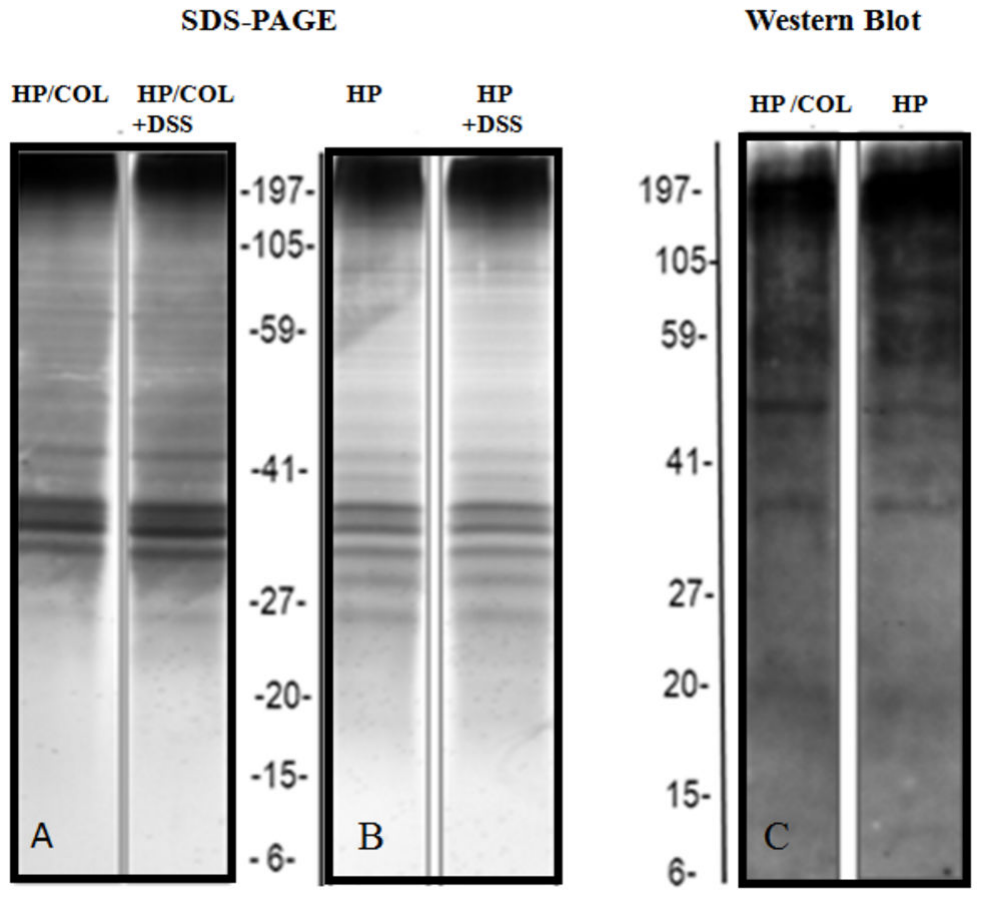
Protein patterns of *H. polygyrus* L4 larvae and *H. polygyrus* antigenic proteins recognized by IgG1 immune sera of BALB/c mice infected with *H. polygyrus*. Protein patterns of L4 nematodes isolated from mice with colitis (HP/COL, A) and from control infection (HP, B) cultured in medium alone and in medium containing 5% DSS (HP+DSS; HP/COL+DSS). L4 antigen was separated by SDS-PAGE in a 4-12% gradient for 40 min at constant 200 V. Gels were silver stained. C: The blot was probed with mouse serum (1:100), followed by horseradish peroxidase-conjugated anti-mouse IgG (1:20000). The representative gel and Western blot immunedetection is shown.

The changes in the worm fitness and protein patterns in mice with colitis were not provoked by DSS directly. Different concentration of DSS *in vitro* did not affect L4 and adult worm survival, egg production by adults or egg hatching. There were no statistically significant differences between results obtained for worms treated directly by DSS and without treatment *in vitro*. The pattern of L4 larvae proteins treated with different concentration of DSS *in vitro* was identical. A representative protein profile of L4 incubated with and without 5% DSS *in vitro* is presented in Figure 6A. However, colitis affected the number of proteins and immunogenic epitopes of parasitic antigens (Figure 6). 

### Identification of immunogenic proteins

**Figure 7:**
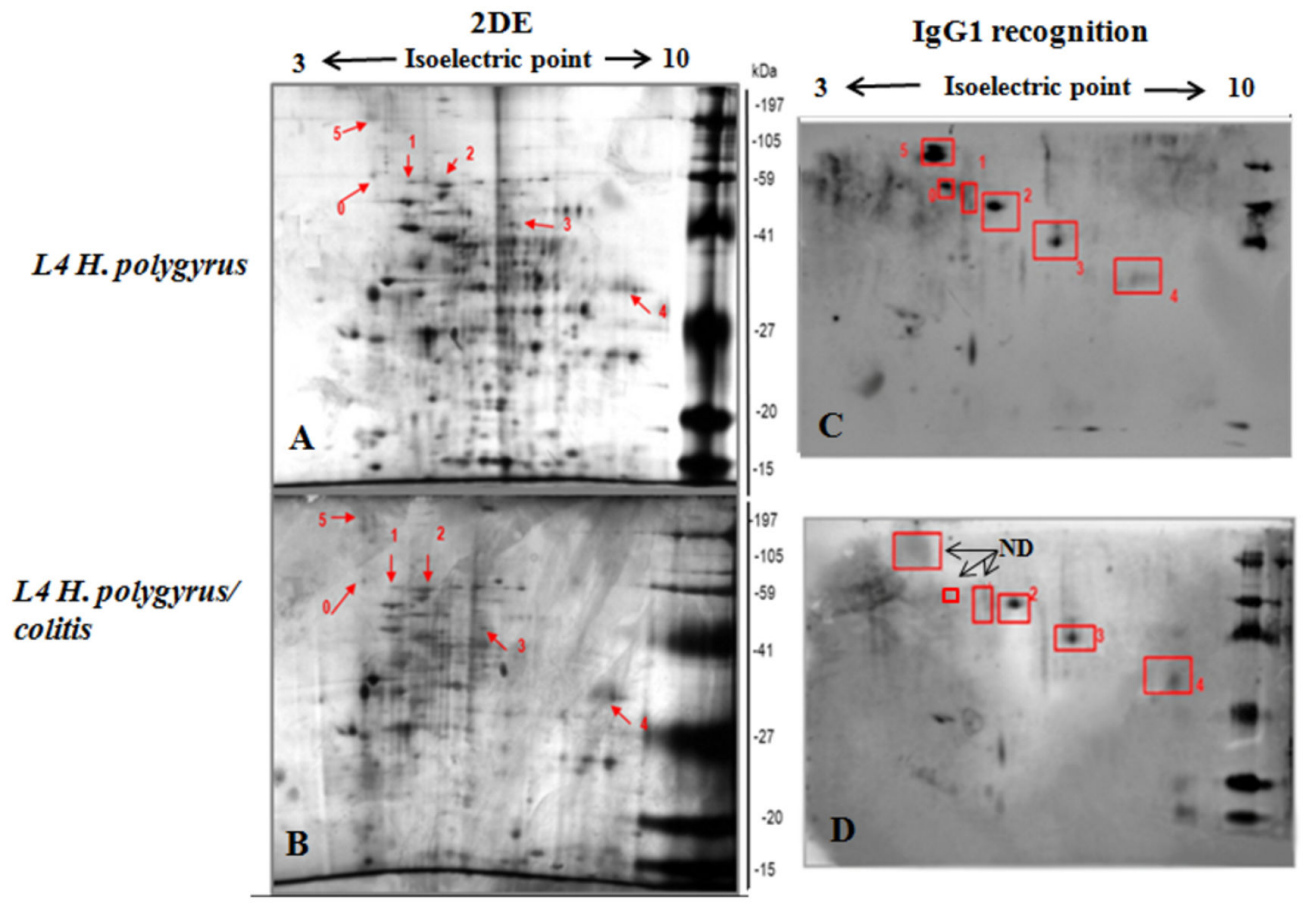
Immuno-reactive spots of *H. polygyrus* L4 isolated from mice with colitis and from control mice. Silver stained two-dimensional polyacrylamide gels of *H. polygyrus* from mice without (A) and with colitis (B). Isoelectric focusing was performed with 30 μg of L4 protein using an IPG strip with a pH range of 3–10. SDS–PAGE was performed on a 12% gel, which was stained with Coomassie brilliant blue colloidal G-250. C. D. The proteins on the 2-D gel were transferred to a nitrocellulose membrane. The blot was probed with mouse serum (1:100), followed by horseradish peroxidase-conjugated anti-mouse IgG (1:20000) and visualized by enhanced chemiluminescence. Spots detected by IgG1 antibody are indicated by arrows and the numbers correlate with: 0- Lev-11 (Isoform 1 of Tropomyosin alpha-1 chain), *C. elegans* (NP_001021695.1); 1 Actin-4 isoform a, *C. elegans* (AAB04575.1), 2- UNC-15, isoform a, (myosin) *C. elegans* (CAB01965.1); 3- EFA-6, isoform c, *C. elegans* (CAM82814.1); 4- ATP synthase alpha and beta subunits, ATP synthase Alpha chain, C terminal *C. elegans* (CAA19429.1 ); 5- FTT-2 isoform a (14-3-3 protein) *C. elegans* (CAA91474.1). Arrows indicate proteins of L4 stage from mice with colitis unrecognized by IgG1 but recognized at L4 stage from control infection.

L4 *H. polygyrus* antigens were separated by 2DE (Figure 7). In this study, spots, mostly located from pH 5 to 9, were detected on global proteome maps of L4 isolated from control mice and mice with colitis using IPG strips. Duplicate gels were blotted onto nitrocellulose and stained with colloidal Coomassie brilliant blue stain. The membrane was probed with the serum of infected mice to visualize immune targets. Six spots of *H. polygyrus* L4 from control infection and three spots from mice treated with DSS were recognized by IgG1 (Table 1). Serum IgG1 did not recognize three spots: actin-4 isoform a, FTT-2 isoform a (14-3-3 protein family) and Lev-11 (isoform 1 of tropomyosin α-1 chain) in L4 from mice with colitis (Figure 7, Table 1). To confirm that these proteins were not recognized, the spots corresponding to the immunoblot were analysed. Spots 0, 1 and 5 were identified as Lev-11, actin-4 isoform a, and 14-3-3 family protein respectively. 2-DE, Western blot and spot analysis were performed in triplicate and identical results were obtained.

**Table 1:** Immuno-reactive protein spots of L4 stage *H. polygyrus* from control infection and mice with colitis and recognition intensity (OD x 10^3^) by IgG1 antibody.

**Protein spot**	**Homologue Accession Number (NCBI**)	**Protein Identified**	**Species**	**IgG1 recognition Spot OD x 10^3^**	
				**HP**	**HP/COL**
0	NP_001021695.1	Protein LEV-11 isoform a	*C. elegans*	89.7	ND
1	AAB04575.1	Actin-4 isoform a	*C. elegans*	132.5	ND
2	CAB01965.1	UNC-15 isoform a	*C. elegans*	185.8	168.9
3	CAM82814.1	EFA-6 isoform c	*C. elegans*	168.9	147.4
4	CAA19429.1	Protein H28O16.1 isoform a (ATP synthase alpha and beta subunits)	*C. elegans*	145.4	164.2
5	CAA91474.1	FTT-2 isoform a (14-3-3 family member)	*C. elegans*	309.3	ND

ND- spots unrecognized by mouse IgG1.

### HPLC profile of L4 antigens

**Figure 8:**
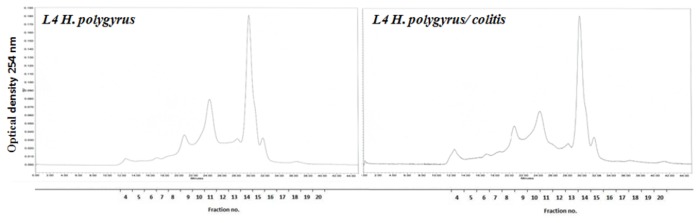
HPLC profiles of peptide preparations obtained by acid elution of L4 antigen from control infection and from mice with colitis. A total of 100 µL of antigen solution was separated on a ProteinPak column and eluted isocratically using PBS (pH 7.4) with flow rate 400 µl/min for 45 min.

L4 somatic extract of both groups yielded 17 major fractions – however, the HPLC profiles revealed variation between antigens. The patterns of HPLC fractions of L4 from colitis-affected intestine differed quantitatively from those obtained from L4 of control infection. Figure 8 shows chromatograms at OD 254nm. 

## Discussion

Many laboratory studies confirm that nematodes prevent and reverse ongoing immune-mediated diseases including IBD, Crohn's disease and colitis, asthma, autoimmune diabetes (type I), rheumatoid arthritis or multiple sclerosis by influencing both innate and adaptive immune reactions. The precise mechanisms of the therapeutic effect of gastrointestinal nematodes are not clearly understood. However, treatment with living helminths *Trichiuris trichiura* and the haematophagous hookworm *Necator americanus* are being used to control immune-mediated IBD in humans [15,16]. Therapy with nematodes or helminths generally is widespread because it is the most effective therapy currently available. It is known that the phenotype of nematodes directly reflects the immune response of the colonized place; different strains of mice respond differently to nematode infection [17]. However, little attention has been paid to the phenotype of nematodes in the disease-affected *milieu*. 

In this study, we showed that the nematode antigenic pattern is actively changed by colitis as soon as 6 days post-infection and the changes in the proteome are associated with differences in nematode fitness. The results indicate that pro-inflammatory changes in the small intestine provoked by colitis result in enhanced worm numbers and growth, increased larval migration, increased male survival and inhibited *per capita* fecundity.

For the study we used the BALB/c mice strain, which is an intermediate responder to *H. polygyrus* infection. This allowed us to demonstrate that the nematode proteome is very malleable in the short-term and is significantly influenced by the intestinal environment, which is changed by colitis. Colitis is restricted to the mucosal layers of the large intestine. *H. polygyrus* worms only inhabit the small intestine; they do not induce inflammation or anatomical changes in the ileum or colon and produce only minimal differences in small intestine cell composition from controls [4]. However, the protective effect of prior *H. polygyrus* L4 larvae at 6 DPI on colitis was associated with inhibited recruitment of leukocytes, especially macrophages, into the inflamed colon mucosa and redirected the leukocytes to the small intestine where the parasite lives [4]. In the present study, we confirmed living *H. polygyrus* therapy redirected the Th2-related response in the small intestine to a Th1/Th17-related response. The leukocyte infiltration into the mucosa and submucosa of the small intestine of mice with colitis at 6 DPI was associated with increased concentration of IL-6, IL-12p70, IL-10, IL-22 MCP-1 and TNF-α, IL-1β, MPO [4] but lower concentration of IL-17A. The monocyte migration into the inflamed mucosa is connected with the chemoattractant MCP-1 as was previously suggested [4]. At 15 DPI in mice with colitis, the production of IL-12p70 and MCP-1 increased and production of regulatory cytokines TGF-β, IL-10 and IL-6 decreased. The Th2-related response is stimulated by recognition of antigens. In mice with colitis, infection provoked shifting to Th1-related responses and higher concentration of specific IgG1 to L4 larvae at 6 DPI but the concentration of specific IgA and IgE was only slightly reduced. 

A major manifestation of immunity to gastro-intestinal nematodes is the failure of infective larvae to establish and mature to adults in the gut. The changes in the small intestine of mice provoked by colitis caused better adaptation of the L3 larvae and worm growth. Only approximately 20% of L3 larvae had not adapted in the gut and were expelled from the intestine. This striking result compares with an establishment of 40% or less in sensitive strains of mice. In mice with colitis, pre-maturation mortality was lower. It was probably connected with the phenomenon of arrested larvae at the L4 (hypobiosis of larvae) and was associated with increased resistance of the hosts to the parasites [18]. The longer maturation and delayed returning to the gut lumen as pre-adults might be responsible for the greater adult size observed. When pre-maturation mortality is low, longer maturation results in longer adults and fecundity. Alternatively, when pre-maturation mortality provoked by host immunity is high, a shorter maturation time produces smaller adults [19].

Sukhedo and Bansemir [20] suggested that changes in the nematode condition could be an adaptive behaviour for more profitable habitats and increased oxygenation. During inflammation in the gastrointestinal tract, there is higher portal and mesenteric blood flow connected with neovascularization of the feeding arteries resulting in increased blood flow to the inflamed tissue [21]. As a consequence of the inflammation in the small intestine, the intestinal position of L4 larvae was altered. Larvae in untreated mice clustered in the duodenum whereas larvae in mice with colitis invaded more distal regions of the small intestine. 

The higher sex ratio (male:female), an indicator of sex-specific survival, of *H. polygyrus* in mice with colitis was also a consequence of the altered immune response. Interestingly, we detected equal survival of males and females at larval and adult stages in mice with colitis. Nematodes have chromosomal sex determination and differential survival between males and females is documented for adult *H. polygyrus* parasites [22]. Adult males are smaller, with a greater surface to volume ratio, than adult females, which may make them more vulnerable to attack by host immune factors under the high-risk environment theory. Alternatively, males in mice with colitis could display their own different, protective molecules according to the results that sex-specific antigens vary between male and female worms [23]. Some molecules presented on males are highly antigenic to mice [22], which may make males more vulnerable.

The immune response in mice with colitis did not affect adult female size but negatively affected the *per capita* fecundity as measured by eggs passed in faeces. Reduction in female worm fecundity as a result of developing or acquired immunity can be measured by reduced faecal egg output, number of eggs in utero or number of newborn larvae during primary infection. The fecundity detected *ex vivo* was naturally varied but lower than in mice with control infection despite the larger size of the female body and the greater number of males. Possibly, nutrient deficiency or factors produced by host cells during colitis are beneficial for nematode survival but not for female egg production. Transfer of live worms from intestine to *in vitro* culture caused recovery of the egg production by females. Another possibility is that the metabolic activity of females could be inhibited by host responses. Different features of the immune response affect different aspects of worm fitness [24]. The immune response of lambs has a greater effect on the faecal egg output of worms than the number of *Teladorsagia circumcincta* [25]. Similarly, immune suppression results in an increase in *Strongyloides ratti* fecundity [26]. 

However, changes in the number of female worms due to expulsion affect the quantity and quality of faeces. Determination of egg production *in vitro* is an independent index of fecundity. The reduction in female worm fecundity of nematodes from mice with colitis during the first 24h *in vitro* confirmed that changes in the small intestine reduced the number of eggs *in utero*. However, incubation of the adult females *in vitro* for 24 hours indicates that decreased production of eggs from each adult female result from changes in the food media [27]. We observed an “explosion” of egg production by females isolated from mice with colitis during next the 48 hours. Further, colitis affected the development of the free-living stages of the next generation. Egg hatching was delayed but the highest viability of L3 larvae was observed *in vitro*. These changes in larvae infectivity and delayed development could be interesting and informative, and are worthy of further investigation.

Immune responses have a major influence on nematode fitness. Murine IgG1 is of particular interest as it has been implicated in immunity to the L4 tissue-dwelling stage of development or earlier. In the natural *H. polygyrus* model, a specific antibody can bind the migrating larvae shortly after inoculation, impairing their penetration and their subsequent migration in the small intestine [28]. However, our results have provided equivocal results. We detected dramatically higher concentrations of L4-specific IgG1 in the small intestine mucous in mice with colitis than untreated mice. However, polyclonal IgG are produced following *H. polygyrus* infection (data not shown) and they limit egg production while parasite-specific IgG1 antibodies affect worm development [29]. Polyclonal antibodies including irrelevant specificities induced better protection than high levels of specific IgG1 antibody [30], but IgG1 limits parasite fecundity. It is possible that the Th2-related response is related to recognition of specific antigens rather than high levels of specific IgG1 antibody.

Changes in the protein pattern of L4 were provoked by the inflammatory reaction in the small intestine. In mice treated with 40kDa DSS, colitis is most prominent in the lower colon. The DSS administered orally is not degraded in the gastrointestinal lumen and DSS may pass intact through the mucosal membrane [31]. However, we additionally excluded a direct influence of DSS on the nematode proteome by electrophoretic analysis of L4 incubated with different concentrations of DSS *in vitro*. 

In this study, six spots of *H. polygyrus* L4 from control infection were recognized by IgG1: actin-4 isoform a, FTT-2 isoform a (14-3-3 protein), Lev-11 (isoform 1 of tropomyosin α-1 chain), UNC-15 isoform a (myosin), EFA-6 isoform c and ATP synthase α and β subunits. Only three spots of L4 isolated from colitis-affected gut were recognized by IgG1 antibody: UNC-15 isoform a (myosin), EFA-6, isoform c and ATP synthase α and β subunits. The proteins not recognized by IgG1 in these larvae were tropomyosin (an actin-associated protein), actin-4 and 14-3-3 protein FTT-2. Spot 3, Lev-11 of *C. elegans* tropomyosin, is a fibrillar protein involved in the contraction of muscle cells, which is included in the actin organization. Spot 1 was matched to actin family member Act-4 of *C. elegans*. These structural proteins are important immunogenic molecules [32]; killing nematode larvae by the host immune response could expose many internal components that are expressed in all life stages of the parasite and some intracellular proteins in the L4, L5 and adult stages might be excreted through certain pathways, which may result in recognition of these structural proteins by the host immune system [32]. Actin is highly conserved throughout evolution and is one of the most abundant proteins in eukaryotic cells. It participates in important cellular functions: muscle contraction, movement of secretory vesicles, cytokinesis, cell division and maintenance of cell shape [33]. The pattern of actin filaments has a definitive role in establishing the annular pattern on the surface of the cuticle. Actin is the core component of the muscle thin filaments, which are highly ordered in sarcomeric structures in striated muscle and, as a component of microvilli, is important to the proper action of nematode intestine. The changes in the immune recognition of actin in L4 presented in our study could influence development. 

Spot 2 was matched to the 14-3-3 protein FTT-2 of *C. elegans*. 14-3-3 protein has been reported from a growing number of helminth parasites. Our results confirmed the strong immunogenic potential of 14-3-3 protein. The native and recombinant hookworm FTT-2 protein expressed in HEK293 cells and *S. mansoni* 14-3-3 protein were recognized by antibodies and induce humoral and cellular immune responses making them potential vaccine antigens [34]. The variability in the proteins of L4 larvae from colitis-affected gut was confirmed in the HPLC analysis.

The complete characterization of these immunogenic molecules in nematodes remains to be performed but some facts are clear. Helminth 14-3-3 protein interacts with the TGF-β Type-1 receptor and enhances TGF-β signalling in the reactivation of tissue-arrested *Ancylostoma caninum* L3 [35]. Recombinant 14-3-3 protein reduces toxicity for the larvae of NO production from activated macrophages *in vitro* [36]. Failure to recognise the FTT-2 isoform of 14-3-3 protein in L4 of mice during colitis could contribute to nematode survival.

Alternative splicing of proteins in nematodes from mice with colitis could lead to changes in the primary amino acid sequence of the protein, sometimes subtle and sometimes quite dramatic, and may affect recognition by serum IgG1. It has been shown to regulate the alternative splicing of its own message, as well as others including α-actin and α-tropomyosin pre-mRNAs [37]. Undoubtedly, differences may arise from the recognition of the same antigen by different antibody classes. In this study, we did not examine changes in protein recognition by IgA and IgE and we did not detect antibody class-switching from IgG-secreting B cells to IgE or IgA but our results clearly show differences in worm number in mice with and without colitis. 

Our experimental studies in the *H. polygyrus* mouse model have advanced our understanding of mucosal immunity acting against intestinal nematodes. Inflammatory bowel diseases such as colitis change the small intestinal cytokine *milieu* and might influence nematode adaptation. The plasticity of the nematode proteome is a consequence of evolutionary adaptation and can be predicted from the success of nematodes in infecting mammalian species. Adaptation of the parasite is beneficial for the host because it inhibits inflammatory disease. However the enhanced adaptation of nematodes in patients with IBD has to be considered. 

## References

[1] Barthlott T, Kassiotis G, Stockinger B (2003) T cell regulation as a side effect of homeostasis and competition. J Exp Med 197: 451–460. doi:10.1084/jem.20021387. PubMed: 12591903. 12591903 PMC2193859

[2] Yazdanbakhsh M, van den Biggelaar A, Maizels RM (2001) Th2 responses without atopy: immunoregulation in chronic helminth infections and reduced allergic disease. Trends Immunol 22: 371–377. PubMed: 11429321. 10.1016/s1471-4906(01)01958-5 11429321

[3] Smith P, Mangan NE, Walsh CM, Fallon RE, McKenzie AN et al. (2007) Infection with a helminth parasite prevents experimental colitis via a macrophage-mediated mechanism. J Immunol 178: 4557–4566. PubMed: 17372014. 17372014 10.4049/jimmunol.178.7.4557

[4] Donskow-Łysoniewska K, Majewski P, Brodaczewska K, Jóźwicka K, Doligalska M (2012) *Heligmosmoides* *polygyrus* fourth stages induce protection against DSS- induced colitis and change opioid expression in the intestine. Parasite Immunol 34: 536–546. doi:10.1111/pim.12003. PubMed: 22889318. 22889318

[5] Donskow K, Drela N, Doligalska M (2011) *Heligmosomoides* *bakeri* antigen rescues CD4 positive T cells from glucocorticoid-induced apoptosis by Bcl-2 protein expression. Parasite Immunol 33: 158-169. doi:10.1111/j.1365-3024.2010.01262.x. PubMed: 21306399. 21306399

[6] Donskow-Łysoniewska K, Brodaczewska K, Doligalska M (2013) *Heligmosomoides* *polygyrus* antigens inhibit the intrinsic pathway of apoptosis by overexpression of survivin and Bcl-2 protein in CD4 T cells. Prion http://dx.doi.org/10.4161/pri.25008. 10.4161/pri.25008 PMC3904318 23787700

[7] Monroy FG, Enriquez FJ (1992) Heligmosomoides polygyrus: A model for chronic gastrointestinal helminthiasis. Parasitol Today 8: 49-54. doi:10.1016/0169-4758(92)90084-F. PubMed: 15463566. 15463566

[8] Liu SK (1965) Pathology of *Nematospiroides* *dubius*. 1. Primary infection in C2H and Webster mice. Exp Parasitol 16: 125–135.

[9] Cypess RH, Lucia HL, Zidian JL, Rivera-Ortiz CI (1977) Heligmosomoides polygyrus: Temporal, spatial, and morphological population characteristics in LAF1/J mice. Exp Parasitol 1 42: 34–43. 10.1016/0014-4894(77)90059-5 862709

[10] Morimoto M, Morimoto M, Whitmire J, Xiao S, Anthony RM et al. (2004) Peripheral CD4 T cells rapidly accumulate at the host: parasite interface during an inflammatory Th2 memory response. J Immunol 172: 2424–2430. PubMed: 14764713. 14764713 10.4049/jimmunol.172.4.2424

[11] Wahid FN, Behnke JM (1992) Stimuli for acquired resistance to *Heligmosomoides* *polygyrus* from intestinal tissue resident L3 and L4 larvae. Int J Parasitol 22: 699–710. doi:10.1016/0020-7519(92)90118-5. PubMed: 1428503. 1428503

[12] Jenkins DC, Ibarra OF (1984) *Nematospiroides* *dubius*: response of the late fourth-stage larva to anthelmintics in vitro. Parasitol Res 70: 395–402. PubMed: 6741225. 10.1007/BF00927827 6741225

[13] Baermann G (1917) Eine einfache methods zur anffinolung von Ankylostomum-(Nematoden)—larven in erdproben. Ned Tijdschr Geneeskd 57: 131–137.

[14] Bizimenyera ES, Githiori JB, Eloff JN, Swan GE (2006) *In* *vitro* activity of *Peltophorum* *africanum* Sond.(Fabaceae) extracts on the egg hatching and larval development of the parasitic nematode *Trichostrongylus* *colubriformis* . Vet Parasitol 142(3): 336-343. 16899339 10.1016/j.vetpar.2006.06.013

[15] Ruyssers NE, De Winter BY, De Man JG, Loukas A, Pearson MS et al. (2009) Therapeutic potential of helminth soluble proteins in TNBS – induced colitis in mice. Inflamm Bowel Dis 15: 491-500. doi:10.1002/ibd.20787. PubMed: 19023900. 19023900

[16] Croese J, O’Neil J, Masson J, Cooke S, Melrose W et al. (2006) A proof of concept study establishing Necator americanus in Crohn’s patients and reservoir donors. Gut 55: 136-137. doi:10.1136/gut.2005.079129. PubMed: 16344586. 16344586 PMC1856386

[17] Morgan C, LaCourse EJ, Rushbrook BJ, Greetham D, Hamilton JV et al. (2006) Plasticity demonstrated in the proteome of a parasitic nematode within the intestine of different host strains. Proteomics 6: 4633–4645. doi:10.1002/pmic.200600068. PubMed: 16858733. 16858733

[18] Balic A, Bowles VM, Meeusen ENT (2000) The immunobiology of gastrointestinal nematodes in ruminants. Adv Parasitol 45: 181–241. doi:10.1016/S0065-308X(00)45005-0. PubMed: 10751941. 10751941

[19] Guinnee MA, Gemmill AW, Chan BHK, Viney ME, Read AF (2003) Host immune status affects maturation time in two nematode species – but not as predicted by a simple life history model. Parasitology 127: 507–512. doi:10.1017/S0031182003003998. PubMed: 14653540. 14653540

[20] Sukhdeo MVK, Bansemir AD (1996) Critical resources that influence habitat selection decisions by gastrointestinal helminth parasites. Int J Parasitol 26: 483–498. doi:10.1016/0020-7519(96)89378-7. PubMed: 8818728. 8818728

[21] Garrelds IM, Heiligers JP, Van Meeteren ME, Duncker DJG, Saxena PR et al. (2002) Intestinal blood flow in murine colitis induced with dextran sulfate sodium. Dig Dis Sci, 47(10): 2231-2236. doi:10.1023/A:1020183110468. PubMed: 12395896. 12395896

[22] Adams JH, East lJ, Monro FG, Washington EA, Dobson C (1987) Stage-specifie antigens of *Nematospiroides* *dubius* Bailys, 1926 (Nematoda: Heligmosomides). J Parasitol 73: 1164–1168. doi:10.2307/3282298. PubMed: 3437354. 3437354

[23] Fengbin Y, Lixin X, Liheng L, Ruofeng Y, Xiaokai S et al. (2010) Immunoproteomic analysis of whole proteins from male and female adult *Haemonchus* *contortus* . Vet J 185: 174–179. doi:10.1016/j.tvjl.2009.05.021. PubMed: 19560953. 19560953

[24] Stear MJ, Bairden K, Duncan JL, Holmes PH, McKellar QA et al. (1997) How hosts control worms. Nature 389: 27–27. doi:10.1038/37898. PubMed: 9288962. 9288962

[25] Stear MJ, Park M, Bishop SC (1996) The key components of resistance to *Ostertagia* *circumcincta* in lambs. Parasitol Today 12: 438–441. doi:10.1016/0169-4758(96)10069-7. PubMed: 15275277. 15275277

[26] Viney ME, Steer MD, Wilkes CP (2006) The reversibility of constraints on size and fecundity in the parasitic nematode *Strongyloides* *ratti* . Parasitology 133: 477–483. doi:10.1017/S003118200600062X. PubMed: 16817996. 16817996

[27] Bansemir AD, Sukhdeo MVK (1994) The food resource of adult *Heligmosomoides* *polygyrus* in the small intestine. J Parasitol 80: 24–28. doi:10.2307/3283340. PubMed: 8308654. 8308654

[28] Liu Q, Kreider T S, Bowdridge Z, Liu Y, Song A (2010) B cells have distinct roles in host protection against different nematode parasites. J Immunol 184: 5213–5223. doi:10.4049/jimmunol.0902879. PubMed: 20357259. 20357259 PMC3729113

[29] McCoy KD, Stoel M, Stettler R, Merky P, Fink K et al. (2008) Polyclonal and specific antibodies mediate protective immunity against enteric helminth infection. Cell Host Microbe 4: 362–373. doi:10.1016/j.chom.2008.08.014. PubMed: 18854240. 18854240

[30] Harris NL, Spoerri I, Schopfer JF, Nembrini C, Merky P et al. (2006) Mechanisms of neonatal mucosal antibody protection. J Immunol 177: 6256–6262. PubMed: 17056555. 17056555 10.4049/jimmunol.177.9.6256

[31] Lorentsen KJ, Hendrix CW, Collins JM, Kornhauser DM, Petty BG et al. (1989) DSS is considered to be a large and negatively charged molecule that cannot easily cross membranes. Ann Intern Med 111: 561-566. doi:10.7326/0003-4819-111-7-561. PubMed: 2476054. 2476054

[32] Fengbin Y, Lixin X, Liheng L, Ruofeng Y, Xiaokai S et al. (2010) Immunoproteomic analysis of whole proteins from male and female adult *Haemonchus* *contortus* . Vet J 185: 174–179. doi:10.1016/j.tvjl.2009.05.021. PubMed: 19560953. 19560953

[33] Fornelio AC, Gonzalez AJ, Caabeiro FR (1995) Actin isoforms in the parasitic nematode *Haemonchus* *contortus* . Parasitol Res 81: 700–702. doi:10.1007/BF00931850. PubMed: 8570588. 8570588

[34] Schechtman D, Winnen R, Tarrab-Hazdai R, Ram D, Shinder V et al. (2001) Expression and immunolocalization of the 14-3-3 protein of *Schistosoma* *mansoni* . Parasitology 123: 573–582. PubMed: 11814044. 11814044 10.1017/s0031182001008769

[35] McGonigle S, Beall MJ, Feeney EL, Pearce EJ (2001) Conserved role for 14-3-3ε downstream of type I TGF-β receptors. FEBS Lett 490: 65–69. doi:10.1016/S0014-5793(01)02133-0. PubMed: 11172812. 11172812

[36] Andrade MA, Siles-Lucas M, Espinoza E, Pérez Arellano JL, Gottstein B et al. (2004) *Echinococcus* *multilocularis* laminated-layer components and the E14t 14-3-3 recombinant protein decrease NO production by activated rat macrophages in vitro. Nitric Oxide 10: 150–155. doi:10.1016/j.niox.2004.03.002. PubMed: 15158694. 15158694

[37] Wollerton MC, Gooding C, Robinson F, Brown EC, Jackson RJ et al. (2001) Differential alternative splicing activity of isoforms of polypyrimidine tract binding protein (PTB). RNA 7: 819–832. doi:10.1017/S1355838201010214. PubMed: 11421360. 11421360 PMC1370133

